# Happiness and air quality: microdata analysis in Indonesia

**DOI:** 10.1186/s41043-024-00517-3

**Published:** 2024-02-07

**Authors:** Novilya Limayani, Erwin Tanur

**Affiliations:** Statistics Indonesia-BPS, Jakarta, Indonesia

**Keywords:** Happiness, IKU, Ordered probit, Health

## Abstract

**Background:**

While economics is growing in Indonesia, its Happiness Index remains steady. Regarding the average concentration of dissolved particles, Indonesia is ranked sixth globally. Many factors can affect happiness. Environmental conditions, especially air quality, are considered to influence individual happiness. Therefore, this research investigates the impact of air quality and health on happiness.

**Methods:**

Data used in this study is the microdata of Indonesia’s Happiness Survey (SPTK) in 2021. With more than 70,000 respondents, the study uses Ordered Probit as an analysis method with subjective happiness as the dependent variable. The independent variables used in this study are air quality, age, gender, housing area per capita, marital status, and health status. All independent variables except age are categorical. The variable of interest, air quality, is coded “1” if the IKU achieves the Strategic Plan’s target of 84.2 and “0” otherwise. IKU is a regional air quality index that combines two substances.

**Results:**

At a significance level of 5%, there is a positive relationship between subjective happiness and air quality. In other words, if air quality (IKU) meets the Strategic Plan target (≥ 84.2), then a person is more likely to have a higher level of happiness. It can be explained more with the marginal effect. The marginal effect concludes that if the target is achieved, the probability of having a lower level of happiness decreases by up to 2.8%, and a very high level of happiness rises by 5.1%. Regarding health status, the rarer someone gets sick, the happier she/he is.

**Conclusion:**

The study finds that air pollution could lower happiness, while better health increases happiness. Therefore, it is important to meet the target of IKU and to improve public health. Some good practices can be adopted to achieve this goal.

## Introduction

Indonesia always targets its economic growth annually. Economic growth is expected to impact the Indonesian population, including individual happiness. Happiness is an essential indicator of life [50]. Happiness is part of subjective well-being (SWB) that can describe economic and social conditions [44]. Happiness is also known as life satisfaction. Since 2013, the Indonesian economy has grown by around 4–5% per year. On the other hand, Indonesia's World Happiness Index [19–27] ranges from 5.1 to 5.3 every year. In other words, even though Indonesia's economy is growing every year, Indonesia's level of happiness remains constant.

Happiness has several causal factors, including physical; social and environmental conditions; and wealth [6]. Liu et al. [39] find a positive relationship between health and happiness. Farhud et al. [9] also found that health and physical attractiveness significantly influence individual happiness. Environmental conditions, especially air quality, are considered to influence individual happiness. Yuan et al. [53] stated that poor air quality had a negative impact on happiness levels. Sanduijav et al. [45] argue that air pollution (PM10) negatively impacts someone’s happiness by concerning his/her family’s health.

Environmental impacts can be measured from several things, for example, air quality. Air quality is an environmental problem not a concern in developing countries [34]. Poor air quality can cause various diseases [7]. The International Agency for Research on Cancer [28] points out that poor air quality can cause chronic diseases such as cancer, heart defects, respiratory problems, and even immune system disorders. Gibson [14] suggests chronic pain can be caused by modifiable environmental factors (e.g., outdoor air quality). Meanwhile, WHO reported that respiratory diseases were the fifth leading cause of death in lower-middle-income countries in 2019. IQAir [29] highlights that South Asia, Southeast Asia, and West Asia had the worst air quality. In their report, IQAir [29] identifies that Indonesia is ranked sixth in the world, regarding the average concentration of dissolved particles, after Bangladesh, Pakistan, Mongolia, Afghanistan, and India.

Mouratidis [42] proposes a framework that links the built environment and SWB. The study describes the environment as a neighborhood. The neighborhood characteristics are categorized as objective,physical and sociodemographic; and perceived. The air pollution is one of the physical characteristics of neighborhoods. Besides, the framework suggests that health is one of the SWB determinants. Thus, environmental conditions, through air pollution, can directly affect physical and mental health. The stressful atmosphere and diseases caused by air pollution ultimately have a negative impact on happiness.

Similarly, Lederbogen et al. [35] argue that there is a possibility that pollution is responsible for mood and anxiety disorders that happen to people who live in the city. Hautekiet et al. [18] state that people tend not to go outside actively when air pollution exists and, hence, are less happy due to a lack of necessities (e.g., moving actively). The evidence reviewed here suggests that air pollution can psychologically impact happiness.

Ferrer-i-Carbonell and Gowdy [11] find that people's awareness of air pollution negatively impacts human well-being. Similarly, Sanduijav et al. [45] argue that people who are satisfied with the quality of their environment have a tendency to be happier. Moreover, Sanduijav et al. [45] state that worries about family health can worsen the effect of air pollution on someone's cardiovascular and respiratory health. In other words, people aware of air pollution and its effects will suffer a double impact on their health since the knowledge also adds some anxiety and respiratory and cardiovascular diseases. In contrast, people who are not aware will suffer from physical diseases. However, according to Steptoe [48], health has a bidirectional relationship with happiness. Happiness has a great role in decreasing the risk of dementia and mild cognitive decline. On the other side, the cognitive decline of the elderly can cause less happiness.

The Indonesian government considers the air quality issue important enough to include this as a target in the Visions and Missions of the President and Vice President of the Republic of Indonesia as outlined in the National Medium Term Development Plan (RPJMN) 2020–2024. The mission is to achieve a sustainable environment, which was then adapted into the Strategic Plan (Renstra) of the Directorate General of Pollution and Environmental Damage Control, Ministry of Environment and Forestry (Ditjen PPKL, KLHK). The fulfillment of this mission is measured by the increasing value of the Environmental Quality Index (IKLH), which consists of the Water Quality Index (IKA), Air Quality Index (IKU), Sea Water Quality Index (IKAL), and Land Cover Quality Index (IKTL). The Directorate General of PPKL is targeting an IKU of 84.2 in 2021.

In Indonesia, one of the air quality measurements is the IKU, one of the IKLH components issued by the Ministry of Environment and Forestry. IKU uses sulfur dioxide (SO_2_) and nitrogen dioxide (NO_2_) as air pollutant parameters (KLHK, nd). These two substances are found in waste from motor vehicles (gasoline), industrial, and diesel vehicles.

## Literature review

A considerable amount of literature has been published on the relationship between happiness and air quality. The Air Quality Index comprises six pollutants: SO_2_, NO_2_, PM10, CO, O_3_, and PM2.5. For example, Xu et al. [52] use the Chinese General Social Survey to find that an increasing natural logarithm of SO_2_ has a negative effect on the subjective happiness of residents. Similar to this, Liu et al. [40] argue that air pollution has a negative effect on happiness. The study examines the direct and indirect effects of pollution on happiness. The happiness is self-reported happiness, while the air pollution is SO_2_ emission. Furthermore, the third model in the study, which includes health and air pollution, shows that health is important regarding the happiness level of a person. So, increasing the level of SO_2_ in the air can decrease the happiness level of an individual. Other research done by Ahumada and Iturra [1] with a variable of air quality, PM 2.5, in Chile; and Goetzke and Rave [15] with SO_2_, NO_x_, and PM10 discover similar findings.

Sanduijav et al. [45] find a negative and significant relationship between air pollution and self-reported well-being. This study uses repeated cross sectional data that matches with air pollution data. Song et al. [47] find that subjective perception of air quality is negatively correlated with self-reported happiness. The negative effect of subjective air pollution is even more significant on sick people and older people, while the effect is unclear for healthy and young people. The study only uses one type of pollutant, PM2.5. According to the Agency of Meteorological, Climatological and Geophysics (BMKG), PM2.5 is an air particle smaller than 2.5 µm. This pollutant is considered to have a more severe effect on human health. The model includes subjective and objective air quality. It should be considered that these two variables measure the same thing, air quality. So, these two variables may have multicollinearity. Furthermore, the AQI information is easily found on the internet, so it can affect one’s perception of air quality. In spite, using the subjective perception from the same happiness survey can be biased.

Zheng et al. [54] find that poor air quality may lower people’s happiness by using sentiment analysis for tweets related to air quality. The air quality variable is PM 2.5, monitored daily. Nevertheless, the proportion of people using tweets must be considered. The question that arises when using social media data is whether the people who tweet represent the population.

Lin et al. [38], focusing on adolescents, discover that early adolescents’ lower happiness levels result from long-term exposure to three pollutants (PM2.5, PM10, and SO_2_), and poor air quality has no impact on changes in happiness levels over time reference. Each pollutant is estimated in different models since the authors find a strong correlation among the pollutants. Despite that, the randomity of the respondents is questionable since the respondents could choose not to participate in the survey.

These results conflict with Khare and Chatterjee [32] who find that air quality is not a determining factor of happiness in Bhopal, India. This study use sentiment analysis of Twitter data, similar with Zheng et al. [54]. However, the independent variables are the urban environment factor that represent other environmental conditions such as public transport, open space, air quality, vegetation index, air pollution, slum, safety and security, and population density. As in Zheng et al. [54], the coverage of the sampel in the study is questionable.

In line with later research, Khasanah and Suryanto [33], with a macro analysis comparing countries in ASEAN, find that air quality had no significant effect on the Happiness Index. This study uses country level data and Human Development Index (HDI), Per Capita Income (PCI), and air pollution as independent variable; and also community happiness as dependent variable. While another research done by Ferreira et al. [10] in Europe, also uses macro data find that the SO_2_ concentration negatively correlate with self-reported life satisfaction.

The existing studies above use only one type of pollutant in their model and have yet to use microdata with Indonesia as a focus. Therefore, this study uses IKU, which combines two types of pollutants as the variable of air quality, to investigate the relationship between air quality and happiness in Indonesia by modeling individual happiness as a function of air quality, health, and demographic variables.

## Research methodology

The data used in this study are microdata from the Happiness Survey (SPTK) 2021 conducted by Statistics Indonesia (BPS). This survey generally aims to measure happiness with subjective assessments of respondents, including material aspects, the meaning of life, and happiness. This survey has been conducted three times since 2014. The respondents from this survey are heads of households or their partners, so questions about happiness relate to individual happiness. However, this study has limitations related to the air quality variables that describe the air quality on the regional (province) level.

Air quality data are obtained from IKU at the provincial level, a component of IKLH calculated by the Ministry of Environment and Forestry in 2021. For information, IKU uses SO_2_ and NO_2_ levels in its calculations. The data of SO_2_ and NO_2_ describe how much of these particles exist in the air. The formula used in the IKU calculation is shown below,

$${\text{IKU}}\, = \,{1}00 - \left( {\frac{50}{{0.9}} \times \left( {I_{EU} - 0.1} \right)} \right).$$

where $I_{EU}$ = European Union’s air quality index (CAQI). So, the higher the IKU, the less pollutants exist in the air, hence the better the air quality. On the other hand, according to WHO, air pollution is a condition when the air is impure because it contains some chemical, physical, or biological agent that changes the structure of the atmosphere.

IKU has the advantage of combining these two air particles. Apart from that, this study classifies these variables into two groups: groups that have achieved the Strategic Plan targets of the Directorate General of PPKL, KLHK and those that have not achieved these targets. The IKU is calculated from data gathered annually. In this case, the data were calculated from 1 January to 31 December 2021. In each regency, the data are gathered from 4 locations: area with heavy traffic, residential area, office area, and industrial area. For your information, Indonesia has more than 500 regencies. At the provincial level, IKU is calculated from the annual average of regencies’ IKU.

The study uses Ordered Probit because the dependent variable, the level of happiness, is ordinal data. The Ordered Probit is used when the dependent variable is ordinal data, not continuous, and does not have the same distance within each classification. For example, the happiness level variable consists of 4 groups. The differences between groups 1 and 2 are not necessarily the same as those between groups 3 and 4. In other words, group 4 does not necessarily have a level of happiness that is four times that of group 1, and vice versa. Furthermore, Ordered Probit is also usually used when the dependent variable has more than two classifications.

Ordered Probit is written as follows [17],

$$\bf y^{*} = {\mathbf{x^{\prime}\beta }} + \varepsilon$$

where $y^{*}$ is the dependent variable, happiness, which has the code 1, 2, 3, 4; x is the independent variable: air quality, age, gender, housing area per capita, marital status, and health status, so that

$$\begin{aligned} & Prob (y = 0|x) = \Phi ( - x^{\prime}\beta ). \\ & Prob (y = 1|x) = \Phi (\mu_{1} - x^{\prime}\beta ) - \Phi ( - x^{\prime}\beta ). \\ & \vdots \\ & Prob (y = J|x) = 1 - \Phi (\mu_{J - 1} - x^{\prime}\beta ).^{{}} \\ \end{aligned}$$

If all probability values are positive, then

$$0 < \mu_{1} < \mu_{2} < \cdots < \mu_{J - 1}$$

With marginal effects as follows:

$$\begin{aligned} & \frac{\partial Prob (y = 0|x)}{{\partial x}} = - \phi (x^{\prime}\beta )\beta , \\ & \frac{\partial Prob (y = 1|x)}{{\partial x}} = - [\phi ( - x^{\prime}\beta ) - \phi (\mu_{{}} - x^{\prime}\beta )]\beta , \\ & \vdots \\ & \frac{\partial Prob (y = J|x)}{{\partial x}} = \phi (\mu_{{}} - x^{\prime}\beta )\beta . \\ \end{aligned}$$

In order to have a relationship between the dependent and independent variables, the Betha needs to be not equal to zero. In the case of ordered probit, the Betha also needs to be not equal. For example, the coefficients of lower categories to all higher categories are not the same in order to be said that the relationship between each pair of the model is different. Furthermore, the coefficients of Ordered Probit are difficult to interpret [17]. However, Daykin and Moffatt [5] provide an example that the sign of the coefficient can be interpreted like ordinary linear regression. Marginal effects show differences in probability (difference in odds) so that Ordered Probit results are more informative.

Table 1 contains information about the operational definition used in this study. The dependent variable in this study is the subjective happiness obtained from question R1501 SPTK 2021: "How happy is [NAME] with life as a whole?". The answer to the question is on a scale of 0–10, where 0 is very unhappy and 10 is very happy. In this study, the scale is classified into four groups based on the classification made by the Official National Statistics [43], United Kingdom, for the well-being survey, especially the life satisfaction question. A scale of 0–4 is categorized as low (code 1), 5–6 is categorized as moderate (code 2), 7–8 is categorized as high (code 3), and 9–10 is categorized as very high (code 4). This paper uses self-reported happiness since it meets the requirements of validity, inclusiveness, reference to presence, sufficiently high signal-to-noise ratio, interpersonal comparability, and availability in valuing public goods [13].

**Table 1:** Operational definition

Variable type	Variable	Operational definition	Data type
Dependent	Happiness	Subjective happiness level	Ordinal with four categories: 1 = Low 2 = Medium 3 = High 4 = Very high
Independent	Air quality	IKU, which is a component of IKLH	Nominal with two categories: 0 = below the 2021 IKU target 1 = meets the 2021 KPI target
Control	Gender	Gender	Nominal with two categories: 0 = female, 1 = male
	Age	Respondent's age at last birthday	Nominal
	Housing area per capita	House area per capita (m^2^) Housing area occupied and used in daily life, divided by the number of household members	Nominal
	Marital status	Marital status at the time of the survey	
	Marry	The respondent's marital status is married	Nominal with two categories: 1 = married 0 = not married
	Divorced	The respondent's marital status is divorced	Nominal with two categories: 1 = divorced 0 = not divorced
	Widowed	The respondent's marital status is widowed	Nominal with two categories: 1 = widowed 0 = not widowed
	Health status	How often do respondents experience health complaints such as gastritis, headaches, coughs, colds, asthma/shortness of breath, diarrhea/wasting water, or other complaints for the last 6 months	
	Usually sick	Respondents usually get sick	Nominal with two categories: 1 = usually get sick 0 = not usually get sick
	Often sick	Respondents often get sick	Nominal with two categories: 1 = often get sick 0 = not often get sick
	Rarely sick	Respondents rarely get sick	Nominal with two categories: 1 = rarely get sick 0 = not rarely get sick
	Never sick	Respondents have never been sick	Nominal with two categories: 1 = never get sick 0 = ever get sick

The air quality variable is the main independent variable. It uses the IKU, divided into two categories: code 0 for the province not achieving the Strategic Plan target and code 1 for the province achieving the Strategic Plan target. IKU is an index that combines SO_2_ and NO_2_. Liu et al. [40] use SO_2_ as an air pollution variable because this substance is the basic ingredient that forms PM 2.5 and has a greater effect on the human body. Meanwhile, NO_2_ is a particulate matter in the air that can be inhaled by humans and can have serious effects both psychologically, genetically, and behaviorally [49].

The control and health variables used in this study are also used by Liu et al. [40]. The control variable aims to minimize type I (false positive) and type II (false negative) errors [37]. In other words, control variables can eliminate bias in the relationship between dependent and main independent variables. Levinson [36] finds that air pollution has a direct relationship with happiness independent of self-reported health. Variable 'health' uses SPTK R701.a "How often does [NAME] experience health complaints (such as fever, headache, cough, cold, shortness of breath) for the last 6 months?", so it is self-reported health of the respondent, and classified into four groups (1, 2, 3, 4).

The variables age, gender, housing size per capita, and marital status are control variables in this study. Bremhorst et al. (2019) use age squared as a variable because of the non-linear effect of age on happiness. One important factor of personal happiness is the size of the house [4, 12]. Marital status determines happiness [8].

## Results and discussion

### Analysis descriptive

Table 2 provides a descriptive analysis of the sample. Around 78% of the sample live in the province that meets the IKU target. There are more than 49% of respondents who are male. Respondents' ages ranged between 14 and 98 years, with an average of 47.43 years. The average housing area per capita is 24.44 m^2^. There are 81% of respondents married, 3% divorced, and 13% are widowed. About 13% of respondents often get sick, 51% rarely get sick, and 35% never get sick.

**Table 2:** Descriptive statistics

Variable type	Variable	Size	Mean	SD	Min	Max
Dependent	Happiness	Subjective happiness level (1–4)	3.11	0.66	1	4
Independent	Air quality	IKU, 0 = below the 2021 IKU target 1 = meets the 2021 IKU target	0.78	0.41	0	1
Control	Gender	0 = female, 1 = male	0.49			
	Age	Respondent's age	47.43	13.50	14	98
	Per capita house area	Housing area per capita (m^2^)	24.44	23.64	0.71	998
	Marital status	Single as a reference				
	Marry	1 = married	0.81	0.39	0	1
	Divorced	1 = divorced	0.03	0.18	0	1
	Widowed	1 = widowed	0.13	0.34	0	1
	Health status	Usually get sick as a reference				
	Often sick	1 = often get sick	0.13	0.34	0	1
	Rarely sick	1 = rarely get sick	0.51	0.34	0	1
	Never sick	1 = never get sick	0.35	0.48	0	1

### Result

Before discussing the Ordered Probit model formed, we will discuss the possibility of multicollinearity between the independent variables of air quality and health. According to Goldberger [16], multicollinearity is a problem that arises if the sample size is very small. The study uses more than 70 thousand samples, and there is no indication of multicollinearity in the model. First, almost all independent variables except the dummy variable, "Widowed", are significant. Second, the standard error of the model is small, and the sign and magnitude of the coefficients do not change in several exercises. Third, the confidence intervals are not too wide. Besides, the variables used in this study are categorical. Therefore, the possibility of high multicollinearity can be ignored.

The results of the analysis using Ordered Probit are shown in Table 3. Almost all independent variables are significant at the 5% level, except for the dummy variable for widowed (*p* value 0.148). The significance means that there is a relationship between the independent and dependent variables; and the relationship for each category of dependent variables to the independent variables are different.

**Table 3:** Ordered probit model

Variable	Coefficient	*p* value	[95% conf. interval]	
Air quality	0.159	0.000*	0.140	0.179
Man	− 0.063	0.000*	− 0.080	− 0.046
Age	− 0.009	0.000*	− 0.012	− 0.005
Age^2^	0.000	0.005*	0.000	0.000
House area per capita	0.003	0.000*	0.003	0.004
Marry	0.362	0.000*	0.308	0.416
Divorced	− 0.127	0.000*	− 0.196	− 0.058
Widowed	0.044	0.148	− 0.016	0.103
Often get sick	0.185	0.046*	0.094	0.275
Rarely get sick	0.315	0.045*	0.226	0.404
Never get sick	0.372	0.046*	0.283	0.461

*Significant at *p* value of 5%

The main independent variable, air quality, has a significant and positive relationship with happiness levels (*p* value 0.000). That means if air quality (IKU) meets the Strategic Plan target (≥ 84.2), then a person is more likely to have a higher level of happiness. The marginal effect results (Appendices 1, 2, 3, 4) are as follows: if IKU reaches the target, then the probability of a person having: a low level of happiness (1) decreases by 0.06% (*p* value 0.000), a medium level of happiness (2) decreases by 2.8% (*p* value 0.000), high level of happiness fell by 1.7% (*p* value 0.000), and very high level of happiness (4) rises by 5.1% (*p* value 0.000).

A positive sign on the health status dummy variable means that someone is more likely to have a higher level of happiness if he/she gets sick less frequently in the past week (all health status dummy *p* value 0.000). For someone who has never been sick, the chance of having: a low level of happiness (1) decreases by 1.9% (*p* value 0.000), a medium level of happiness (2) decreases by 7.3% (*p* value 0.000), a high level of happiness (3) decreases by 1.6% (*p* value 0.000), and very high level of happiness (4) rises by 10.9% (*p* value 0.000).

A man is more likely to be unhappy than a woman (*p* value 0.000). The older person is more likely to have a lower level of happiness (*p* value 0.000). The larger the housing area per capita, the greater the probability of a person having a higher happiness level (*p* value 0.000). Married people are more likely to have a higher level of happiness than someone single (*p* value 0.000). Meanwhile, divorced people are more likely to have lower levels of happiness than those who are single (*p* value 0.000).

### Discussion

This study examines the relationship between happiness and air quality using SPTK data with a total of 74,684 respondents in Indonesia. This study finds that air quality has a significant positive impact on happiness. In other words, when the IKU reaches the Strategic Plan target, people are more likely to have a higher level of happiness than those living in a province that has not yet reached the IKU target. It is understandable since poor air quality negatively impacts emotional levels and mood, decreasing individual happiness. [42].

These results are in line with previous research conducted in China by Liu et al. [40], Sanduijav et al. [45], Xu et al. [52], Zheng et al. [54], and in Chile by Ahumada and Iturra [1], and in Germany by Goetzke and Rave [15] that uses an actual measure of air quality. Note that the sign of the coefficient of air quality seems different from those in Liu et al. [40].

The reason is that both studies use different terms of air quality. While this study uses the term air quality, Liu et al. [40] use air pollution, so the direction of both are different. The higher the coefficient of air quality, the cleaner the air is, while the higher the coefficient of air pollution, the dirtier the air. All the research mentioned above uses individual data and self-reported happiness. Using subjective air pollution, Song et al. [47] also find that the more severe air pollution is, the less happy people are. In addition, the happiness data for some research are obtained differently. For example, Zheng et al. [54] use daily public express happiness data via social media, while others use self-report data from surveys.

On the contrary, this study’s results differ from that of Khasanah and Suryanto [33], which find no relationship between air quality and the Happiness Index. Khasanah and Suryanto [33] use macro data from ASEAN countries with the PM 2.5 level as air pollution variables and the Happiness Index as their dependent variable. In addition, the observation unit in the study is country, while this study uses individual data as unit observation. In other words, the study uses community data, not individual data.

Another result of this study is the marginal effect from The Ordered Probit analysis model that complements information from previous research. The probability of a person having a higher level of happiness is higher when he/she lives in a province that has met the IKU target. The marginal effects show that when the IKU target is achieved, the differences of probabilities in the happiness level of “very high” rise significantly to 5.1%. In contrast, the probability of the lower level of happiness falls. This result confirms that when the air quality is better, marked by the achievement of IKU’s target, people will be happier.

The study also finds that health status has a positive impact on happiness. As noted by MacKerron and Mourato [41], good health can increase life satisfaction. Similar results are also found by Levinson [36], who used General Social Survey (GSS) data in several regions in America. Low levels of health have a negative impact on a person's happiness. This finding is also confirmed by Liu et al. [39], with China as the focus study. However, health and happiness may have a bidirectional relationship [48].

## Conclusions and recommendations

This study analyzes the relationship between happiness, air quality, and health. Poor air quality decreases happiness, and good health increases happiness. On the other hand, it is possible that health and happiness have a two-way causal relationship. It is important to achieve the target of IKU by controlling air pollution and also improving public health since it can increase people's happiness.

A policy can be evaluated with the right and suitable indicators [44]. In the case of the environment, emissions and pollution can be used as indicators. Nevertheless, happiness can describe how a policy impacts the population [44]. This study shows that air quality can affect happiness. For that reason, the government should take the right steps to increase air quality in Indonesia for a better life.

United Nations Environment Programme [51] stated in an article that some countries have succeeded in enhancing air quality. Here are the brief ideas from that article. Bogota aggressively switches to electric public transportation and also emphasizes the importance of using bicycles as transportation. Warsaw installs air sensors across the city in order to build a database that will be used in creating policies related to air pollution control. Warsaw also gradually reduced coal in producing energy. South Korea will have banned diesel vehicles as public transportation in 2025. Accra prepares the disposal system, transportation, and sustainable energy and educates its citizens on handling rubbish. Bangkok creates more green spaces with a project of 11 parks to reduce pollution. Bangkok also built a 15 km *greenway* to reduce the dependency on private vehicles.

Indonesia can adopt all the strategies explained above. Indonesia’s air pollution sources are emissions from traffic, biomass burning, dust, and also forest and peat fires [31]. The air pollution is most noticed in Jakarta [3]. Although Jakarta has Transjakarta, the BRT introduced in 2004 [2], LRT, and MRT, the air pollution is even worse. According to IQ Air [30], although there were some improvement in air quality in Southeast Asia, Indonesia still has the worst pollution in 2021. Therefore, Indonesia must adopt some strategies to manage air pollution.

In order to control air pollution that comes from the transportation sector, Indonesia, especially in big cities, has to make a policy that limits the ownership of private cars while at the same time having a great public transportation system. Sefriyadi et al. [46] suggest several policies that can be adopted, especially in big cities. The policies include restrict garage capacity, better governance of driving license mechanism, enhance quality of public transportation in terms of safety and comfort, increase personal transport tax, and introduce car quota system. In addition, the strategies done by Bogota (electrifying public transportation) and South Korea (banning diesel public transportation) can also be implemented. To decrease pollution from biomass burning, Indonesia can adopt Warsaw’s strategy to reduce the usage of coal in producing energy.

Due to this study limitation, future studies can use real-time PM2.5 or PM10 data as an air quality variable in smaller areas so that the air quality variable will be moving across individuals for the future study to be more accurate. This study uses self-reported health as a health variable. However, future studies can use the health status related to respiratory diseases and consider it a mediator in the relationship between happiness and air pollution. Furthermore, future studies can also examine how long-term pollution affects the changes in happiness levels between regions since it is also possible that one is accustomed to air pollution after living in the area for an amount of time.

## Data Availability

The data supporting this study's findings are available on Statistics Indonesia (BPS) via SILASTIK. However, restrictions apply to the availability of these data, which were used under license for the current study are not publicly available. The data are available if one submits the request to Statistics Indonesia (BPS) via SILASTIK.

## Appendix 1

See Table 4.

**Table 4:** Marginal effect: low level of happiness (1)

Variable	Coefficient	*P* value	[95% conf. interval]	
Air quality	− 0.006	0.000	− 0.007	− 0.005
Man	0.002	0.000	0.002	0.003
Age	0,000	0.000	0,000	0,000
House area per capita	0,000	0.000	0,000	0,000
Marry	− 0.014	0.000	− 0.016	− 0.012
Divorced	0.005	0.000	0.002	0.008
Death divorce	− 0.002	0.149	− 0.004	0.001
Often sick	− 0.011	0.001	− 0.018	− 0.005
Rarely sick	− 0.017	0.000	− 0.024	− 0.011
Never sick	− 0.019	0.000	− 0.026	− 0.013

## Appendix 2

See Table 5.

**Table 5:** Marginal effect: medium level of happiness (2)

Variable	Coefficient	*P* value	[95% conf. interval]	
Air quality	− 0.028	0.000	− 0.032	− 0.025
Man	0.011	0.000	0.008	0.014
Age	0.001	0.000	0.001	0.001
House area per capita	− 0.001	0.000	− 0.001	− 0.001
Marry	− 0.064	0.000	− 0.074	− 0.055
Divorced	0.023	0.000	0.010	0.035
Death divorce	− 0.008	0.149	− 0.018	0.003
Often sick	− 0.039	0.000	− 0.058	− 0.019
Rarely sick	− 0.063	0.000	− 0.083	− 0.044
Never sick	− 0.073	0.000	− 0.092	− 0.053

## Appendix 3

See Table 6.

**Table 6:** Marginal effect: high level of happiness (3)

Variable	Coefficient	*P* value	[95% conf. interval]	
Air quality	− 0.017	0.000	− 0.019	− 0.014
Man	0.007	0.000	0.005	0.008
Age	0.000	0.000	0.000	0.000
House area per capita	0.000	0.000	0.000	0.000
Marry	− 0.038	0.000	− 0.044	− 0.032
Divorced	0.013	0.000	0.006	0.021
Death divorce	− 0.005	0.148	− 0.011	0.002
Often sick	0.000	0.976	− 0.004	0.004
Rarely sick	− 0.010	0.000	− 0.014	− 0.006
Never sick	− 0.016	0.000	− 0.021	− 0.012

## Appendix 4

See Table 7.

**Table 7:** Marginal effect: very high level of happiness (4)

Variable	Coefficient	*P* value	[95% conf. interval]	
Air quality	0.051	0.000	0.045	0.058
Man	− 0.020	0.000	− 0.026	− 0.015
Age	− 0.001	0.000	− 0.001	− 0.001
House area per capita	0.001	0.000	0.001	0.001
Marry	0.116	0.000	0.099	0.134
Divorced	− 0.041	0.000	− 0.063	− 0.018
Death divorce	0.014	0.148	− 0.005	0.033
Often sick	0.050	0.000	0.027	0.073
Rarely sick	0.090	0.000	0.068	0.112
Never sick	0.109	0.000	− 0.021	− 0.012
